# An Improved DINEOF Algorithm for Filling Missing Values in Spatio-Temporal Sea Surface Temperature Data

**DOI:** 10.1371/journal.pone.0155928

**Published:** 2016-05-19

**Authors:** Bo Ping, Fenzhen Su, Yunshan Meng

**Affiliations:** 1 School of Remote Sensing and Information Engineering, Wuhan University, Wuhan 430079, China; 2 Laboratory of Resource and Environmental Information System, Institute of Geographic Sciences and Natural Resources Research, Chinese Academy of Sciences, Beijing 100101, China; 3 University of Chinese Academy of Sciences, Beijing 100049, China; Glasgow University, UNITED KINGDOM

## Abstract

In this study, an improved Data INterpolating Empirical Orthogonal Functions (DINEOF) algorithm for determination of missing values in a spatio-temporal dataset is presented. Compared with the ordinary DINEOF algorithm, the iterative reconstruction procedure until convergence based on every fixed EOF to determine the optimal EOF mode is not necessary and the convergence criterion is only reached once in the improved DINEOF algorithm. Moreover, in the ordinary DINEOF algorithm, after optimal EOF mode determination, the initial matrix with missing data will be iteratively reconstructed based on the optimal EOF mode until the reconstruction is convergent. However, the optimal EOF mode may be not the best EOF for some reconstructed matrices generated in the intermediate steps. Hence, instead of using asingle EOF to fill in the missing data, in the improved algorithm, the optimal EOFs for reconstruction are variable (because the optimal EOFs are variable, the improved algorithm is called VE-DINEOF algorithm in this study). To validate the accuracy of the VE-DINEOF algorithm, a sea surface temperature (SST) data set is reconstructed by using the DINEOF, I-DINEOF (proposed in 2015) and VE-DINEOF algorithms. Four parameters (Pearson correlation coefficient, signal-to-noise ratio, root-mean-square error, and mean absolute difference) are used as a measure of reconstructed accuracy. Compared with the DINEOF and I-DINEOF algorithms, the VE-DINEOF algorithm can significantly enhance the accuracy of reconstruction and shorten the computational time.

## Introduction

Satellite-derived sea surface temperature (SST) data from infrared observations have been widely used in oceanography due to their extensive coverage, in time and space. However, these SST data are often influenced by the presence of clouds in the atmosphere, malfunctions in the satellite or images noises, which can cause missing data. Additionally, the loss of data may reach a high percentage in some periods. Undoubtedly, a complete data set is desirable or even essential for many applications using satellite-derived data. There are currently many different methods, such as spline interpolation [1] and optimal interpolation (OI) [2–4], that have been developed to deal with recovery of missing data.

In 2003, Beckers and Rixen [5] presented the Data INterpolating Empirical Orthogonal Functions (DINEOF) algorithm which is a parameter-free technique based on an iterative EOF decomposition to calculate missing data in satellite data sets without requiring a priori knowledge about statistics of the full dataset. Recently, the researches on DINEOF algorithm mainly contain: satellite-derived oceanographic images reconstruction for further analyses; the algorithm accuracy improvement; the error analysis of DINEOF algorithm; outlier detection. [6–18]. This study focuses on the algorithm improvement.

In the ordinary DINEOF algorithm, the original dataset is first stored in a spatio-temporal matrix with m×n dimensions and the temporal and spatial average is removed from the data. The first EOF mode is then calculated by using Singular Value Decomposition (SVD) technique, which is used to infer a new estimate for the missing data. This procedure is repeated until convergence is obtained for the values given to the missing data with the first EOF mode. Subsequently, the number of EOFs increases one by one and for each EOF mode, the whole reconstruction will be operated again until convergence. Then by using cross-validation technique, the optimal EOF mode will be determined. Finally, the reconstruction procedure will be performed again based on the optimal EOF mode until the convergence is reached.

In the ordinary DINEOF algorithm, due to the optimal EOF mode determination, the reconstruction procedure will be first repeated until convergence for each EOF mode. Generally the optimal EOF is unknown before the reconstruction, so the maximum number of EOFs is commonly predefined larger than the optimal EOF mode. Hence, it definitely takes much computational time to determine the optimal EOF. For example, if the maximum number of EOFs is predefined as 100, then the reconstruction will be first iteratively operated at least 100 times to obtain the optimal EOF. Then, after obtaining the optimal EOF, the whole iterative process also needs to be performed again to acquire the final result. Therefore, the DINEOF algorithm seems to be inefficient. On the other hand, the initial spatio-temporal matrix with missing data is reconstructed based on the fixed optimal EOF in the ordinary DINEOF algorithm, however, this optimal EOF mode may be not the best one for some reconstructed matrices in the intermediate steps, which means the accuracy of reconstruction may be probably influenced.

Therefore, an improved DINEOF algorithm is presented in this study. In the improved algorithm, the optimal EOFs are variable and should be determined by using cross-validation technique based on the reconstructed matrices in the intermediate steps, hence, the improved DINEOF algorithm is called VE-DINEOF (variable EOFs DINEOF) in this study. In addition, the whole iteration procedure is only implemented once, which can obviously enhance the algorithm efficiency.

Ping et al. (2015) [14] proposed an improved DINEOF algorithm (I-DINEOF) that reconstructed the gappy spatio-temporal SST data based on local optimal EOF mode determined by SST data of subarea. The I-DINEOF algorithm has been proved to be valid to enhance the recovery accuracy and therefore it will be used to make a comparison with the proposed VE-DINEOF algorithm. Compared with VE-DINEOF algorithm, the cross-validation points are not necessary to set aside for the optimal EOF mode determination in the I-DINEOF algorithm. However, similar with the ordinary DINEOF algorithm, before the local processing in the I-DINEOF algorithm, the gappy spatio-temporal matrix requires to be reconstructed based on every EOF mode, which means the I-DINEOF algorithm will take more time. Actually, these two algorithms both aim to enhance the ordinary DINEOF algorithm by changeable optimal EOF mode but they reach this objective in two different ways. The I-DINEOF algorithm uses the local processing to find the local optimal EOF mode, and the VE-DINEOF algorithm uses the changeable and optimal EOF mode for each intermediate reconstructed matrix in the iteration processing.

In the following, Section 2 introduces the data set. The main algorithm introduction is described in Section 3. The validation of VE-DINEOF algorithm is discussed in Section 4. Section 5 discusses some details of VE-DINEOF algorithm. Finally, conclusions are given in Section 6.

## Data Set

**Fig 1:**
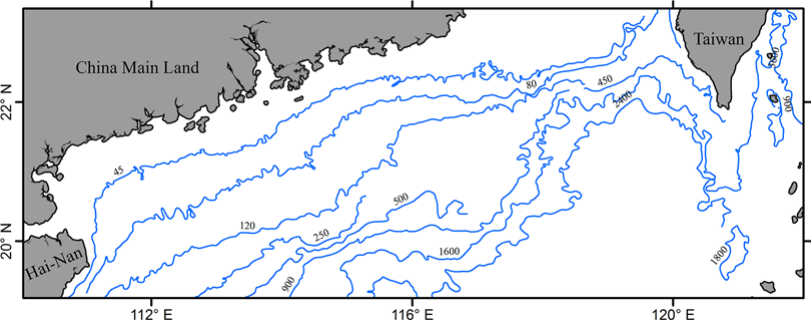
The northern South China Sea and its bathymetry (depth in meters).

In this study, daily 4-km night-time Advanced Very High Resolution Radiometer (AVHRR) Pathfinder Version 5.2 (PFV5.2) SST data of the northern South China Sea (NSCS) spanning from 1 January 2011 to 31 December 2012 were used in this work. The geographic area covers 110–122°E and 19–23°N and Fig 1 provides a general view of the domain of interest with isobaths in meters and borders. The data are freely available from the US National Oceanographic Data Center and GHRSST (http://pathfinder.nodc.noaa.gov). The PFV5.2 data are an updated version of the Pathfinder Version 5.0 and 5.1 collection described in [19]. The initial size of the SST data set is 100×290 pixels and 731 images. For PFV5.2 SST dataset, a quality level, ranging from 0 (worst) to 7 (best), is assigned to every pixel and pixels with a quality level of 3 or less are flagged as missing data in this paper.

**Fig 2:**
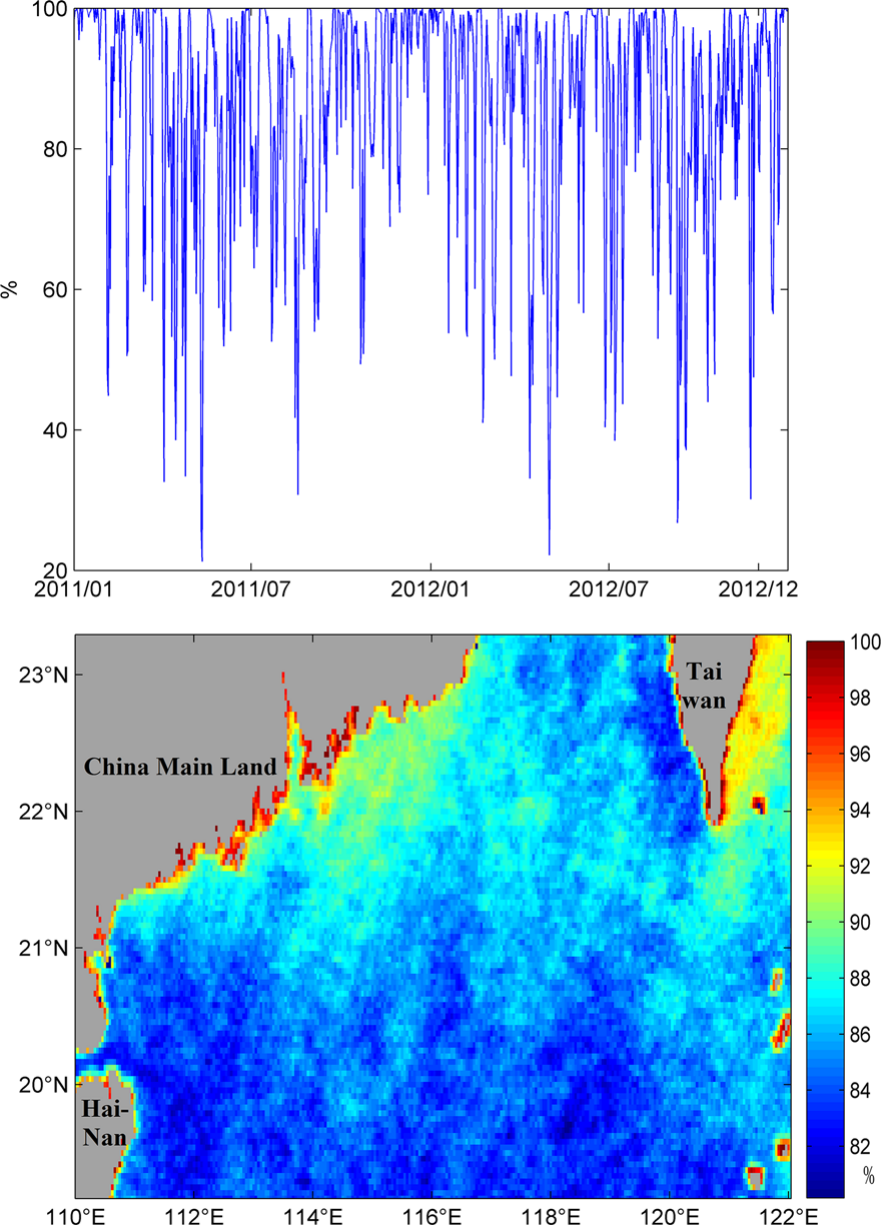
Top panel: temporal variation of missing data percentage in the NSCS. The year/month labels show the 1st of corresponding year/month. Bottom panel: spatial variation of missing data percentage in the NSCS.

The average loss of this data set is about 86%. Temporal variability of total missing data presents an irregular distribution with minimum average missing data of about 40% to 60% and maximum average missing data of more than 90% (Fig 2). Spatially, regions with the highest missing data percentage are the coastal waters of China Main Land and eastern waters of Taiwan, with an average loss higher than 90%. In the rest of the NSCS, the average missing data varies from 80% to 90%. Typical missing data sizes are comparative large, covering most of the experimental area at a time.

Images containing less than 5% of existing data do not provide useful information and might affect the final result [9]. Hence, prior to VE-DINEOF treatment, it was chosen to eliminate each image holding less than 5% of the expected data. This reduced the number of exploitable images from 731 to 408. The same elimination criterion was applied in time, excluding thus from the study all pixels holding less than 5% of valid data through the temporal dimension. Land points were not used in this methodology, so the final spatial size is 22770 (out of 29000) and a subset of 408 images was kept for reconstruction.

## Methods

### 3.1 The VE-DINEOF Algorithm

Beckers and Rixen (2003) have presented the DINEOF algorithm which is a self-consistent method for the reconstruction of missing data in oceanographic data sets. In this study, the VE-DINEOF algorithm was applied as follows.

1. The original dataset was first stored in the initial m×n matrix, where m is the number of pixels and n is the number of images (22770×408 in this study). This matrix contains both existing and missing pixels. A random 3% of the valid data (67925 pixels in this study) in the matrix were set to 0 in the initial matrix, and these values set aside (deemed as missing data in the reconstruction process) for use in cross-validation. Then the corresponding spatio-temporal mean of the initial matrix was subtracted from the initial matrix. Missing data points were set to 0 (unbiased guess). This processed matrix was designated as X.
2. The EOF decomposition was computed by using the Singular Value Decomposition (SVD) technique. The SVD method can be defined as Eq (1):
  $$X=USV^{T}$$ (1)
  Where **U** with dimension m×r contains on each of its columns one of the spatial patterns of the EOFs; the pseudo-diagonal matrix **S** with dimension r×r signifies the singular values; **V** with dimension n×r denotes the temporal EOFs. The value r is the rank of the matrix **X**, with r ≤min(m,n). The singular values and the respective vectors are sorted to decreasing order.
3. Only the most significant spatial and temporal EOFs are necessary for the reconstruction methodology so that the truncated reconstruction **X**_**r**_ is the best estimate of the field, which can be calculated based on Eq (2):
  $$X_{r}=U_{N}S_{N}V_{N}^{T}$$ (2)
  Where **U**_**N**_ is a m×N matrix with N columns containing the first N spatial EOFs, **V**_**N**_ is a n×N matrix with N columns containing the first N temporal EOFs and **S**_**N**_ a diagonal matrix of size N×N containing the first N singular values.
4. In contrast with the ordinary DINEOF algorithm, the N value in VE-DINEOF algorithm is changeable and needs to be determined based on every reconstructed matrix generated in the intermediate steps by using cross-validation technique. The first SVD decomposition was performed on **X** and the RMSEs for all EOFs between the original values and reconstructed values at the cross-validation points were calculated. To save computational time and enhance algorithm efficiency, the maximum number of EOFs was set to 300 in this study. By finding the smallest RMSE, the optimal EOF can be determined and then the missing data could be calculated by Eq (2) with the optimal EOF mode. Then gappy data were reconstructed with values for the existing data were kept for original values and values for the missing points were replaced by the reconstructed values obtained from the optimal EOF.
5. Then, if the convergence was not reached, the number of SVD decompositions increased one by one until the convergence was reached. After each SVD decomposition, the optimal EOF was determined again and the matrix was reconstructed by using the updated optimal EOF. The iteration continued to produce improved missing data until the changes observed at the cross-validation points between one iterative cycle and the next one were insignificant. The convergence criterion was reached when the RMSE between the two iterations at the cross-validation points became lower than a threshold value of 1.0e^-3^. To avoid the iterative death loop and save computational time, the greatest number of SVD decompositions was predefined as 100.

### 3.2 Validation of Reconstruction Accuracy

Four parameters including Pearson correlation coefficient (r), signal to noise ratio (SNR), RMSE and mean absolute difference (MAD) were calculated to validate the accuracy of the VE-DINEOF algorithm. These statistical parameters obtained from the original values and the corresponding reconstructed values for the valid data can be used to evaluate the accuracy of the reconstruction.

The RMSE and MAD are defined as follows:

$$RMSE=\sqrt{\frac{\sum {\left(S-I\right)}^{2}}{n}},MAD=\frac{\sum \left|S-I\right|}{n}$$ (3)

Where S is original SST value, I is the reconstructed SST value and n is the number of match-up samples. The SNR is the ratio of standard deviation of the reconstructed values and the standard deviation of the errors (difference between original values and reconstructed values) for the valid data points.

## Results

To better validate the VE-DINEOF algorithm, the I-DINEOF algorithm was also added in this paper to make a comparison with the VE-DINEOF algorithm. In the I-DINEOF algorithm, the size of subarea is a significant parameter for reconstruction. On the one hand, the size should not be too large so as to acquire the best EOF mode for the subarea; on the other hand, there must be some existing points to determine the optimal EOF mode and some missing points to fill in in each subarea. In this paper, the proportion of missing data in the dataset is higher than that in Ping’s paper (2015) due to daily SST data usage, so the size of subarea was set as 25 pixels. The size setting is subjective to some extent and some further researches need to be done to solve this problem. The maximum number of EOFs was set to 300, which is the same with the VE-DINEOF algorithm.

The ordinary DINEOF, I-DINEOF and VE-DINEOF algorithms were directly performed on PFV5.2 SST data, predefined in Section 2. SNR, r, RMSE and MAD from the reconstructed and original values for the existing points were used as a measure of reconstructed accuracy. Table 1 summarizes the four validation parameters of the DINEOF, I-DINEOF and VE-DINEOF methods. Table 1 shows that compared with the ordinary DINEOF algorithm, the VE-DINEOF algorithm can increase r and SNR from 0.9943 and 11.0682 to 0.9987 and 19.9641, respectively; decrease RMSE and MAD from 0.2773°Cand 0.1515°C to 0.1303°C and 0.0155°C, respectively. Therefore, these results present a significant improvement in reconstructed accuracy with the VE-DINEOF algorithm. Moreover, the VE-DINEOF costs less computational time than DINEOF algorithm.

**Table 1:** Reconstructed results using DINEOF and VE-DINEOF methods.

	r	SNR	RMSE (°C)	MAD(°C)
DINEOF	0.9943	11.0682	0.2773	0.1515
I-DINEOF	0.9964	17.1149	0.2215	0.1631
VE-DINEOF	0.9987	19.9641	0.1303	0.0155

Additionally, compared with I-DINEOF algorithm, the VE-DINEOF can increase r and SNR from 0.9964 and 17.1149 to 0.9987 and 19.9641, respectively; decrease RMSE and MAD from 0.2215°C and 0.1631°C to 0.1303°C and 0.0155°C, respectively. The reason for that is probably the size of subarea. Ideally, one missing point can be best reconstructed by its corresponding optimal EOF mode. With the increasing of the size of subarea, the number of missing points in one subarea will become large and hence the optimal EOF mode for that subarea is a compromise, which means the optimal EOF may be not the best one for some missing points in that subarea. On the other hand, the size of the subarea cannot be set too small, because there must be enough existing points to determine the local optimal EOF mode and some missing points to fill in. Therefore, if the size of subarea can be better determined, we believe the accuracy of I-DINEOF algorithm may be boosted though probably more researches should be done for the size setting procedure. Similar with the ordinary DINEOF algorithm, the reconstructed matrix based on every EOF mode should be calculated first in the I-DINEOF algorithm, so the efficiency of VE-DINEOF algorithm is greatly enhanced.

Even though the accuracy of I-DINEOF algorithm is lower than VE-DINEOF algorithm, compared with the ordinary DINEOF algorithm, these two algorithms can both get better results.

**Fig 3:**
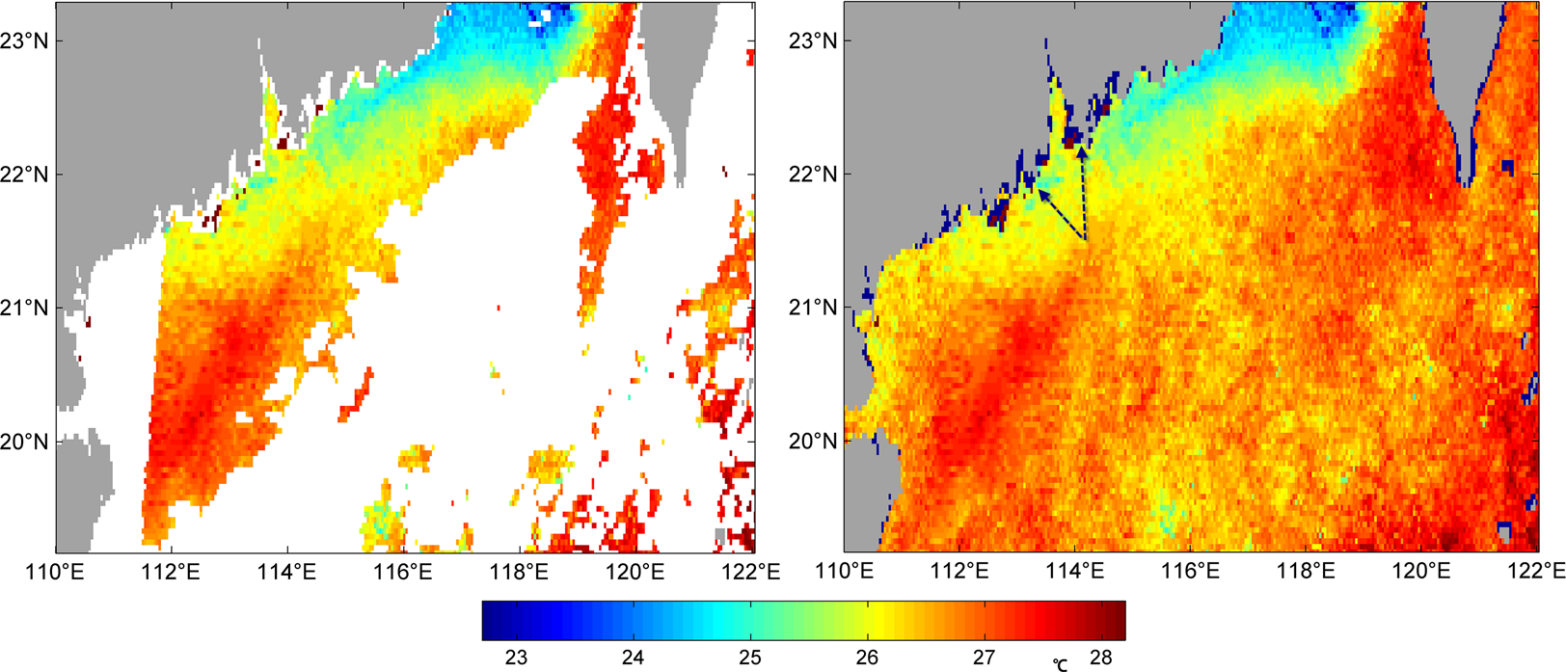
Left panel: original gappy image for 25^th^ October, 2011; right panel: its reconstruction image. The dark blue points shown by arrows represent the points with less than 5% good data in the temporal dimension.

**Fig 4:**
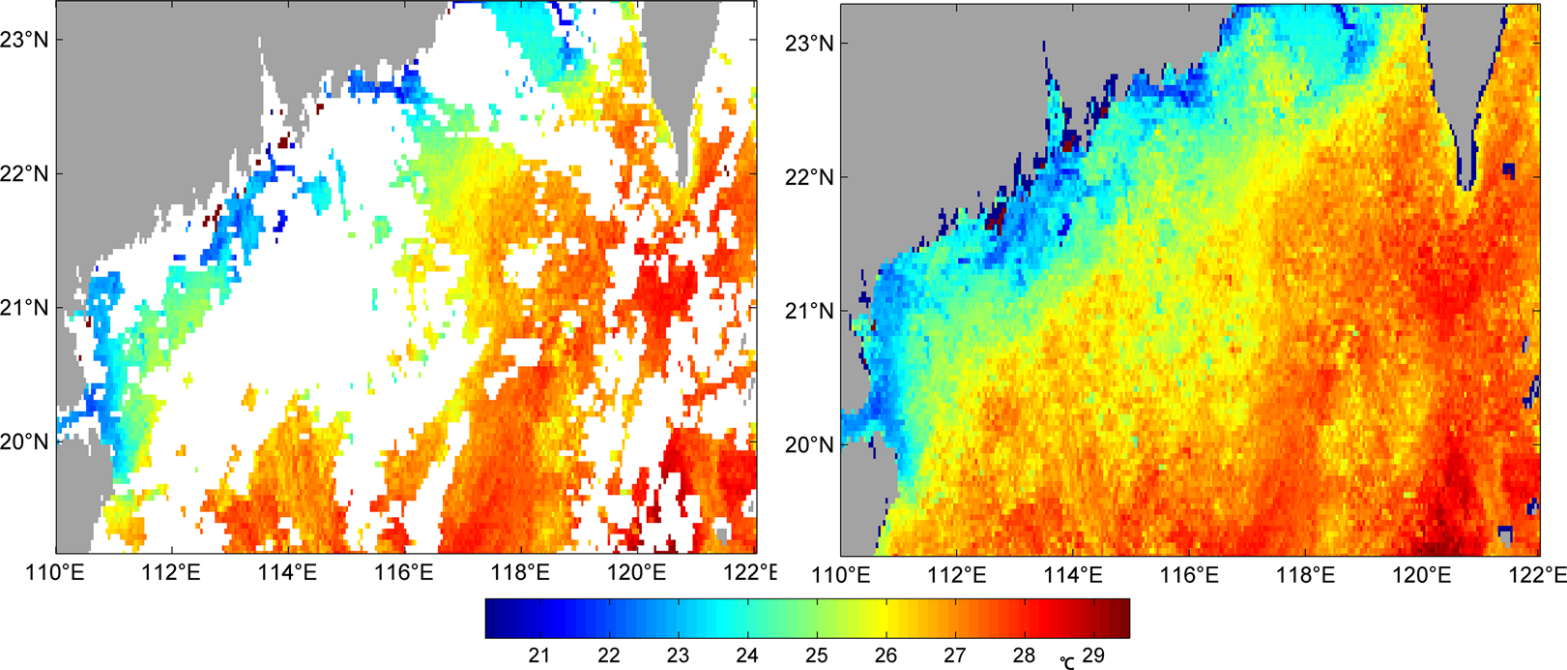
Left panel: original gappy image for 12^th^ April, 2012; right panel: its reconstruction image.

**Fig 5:**
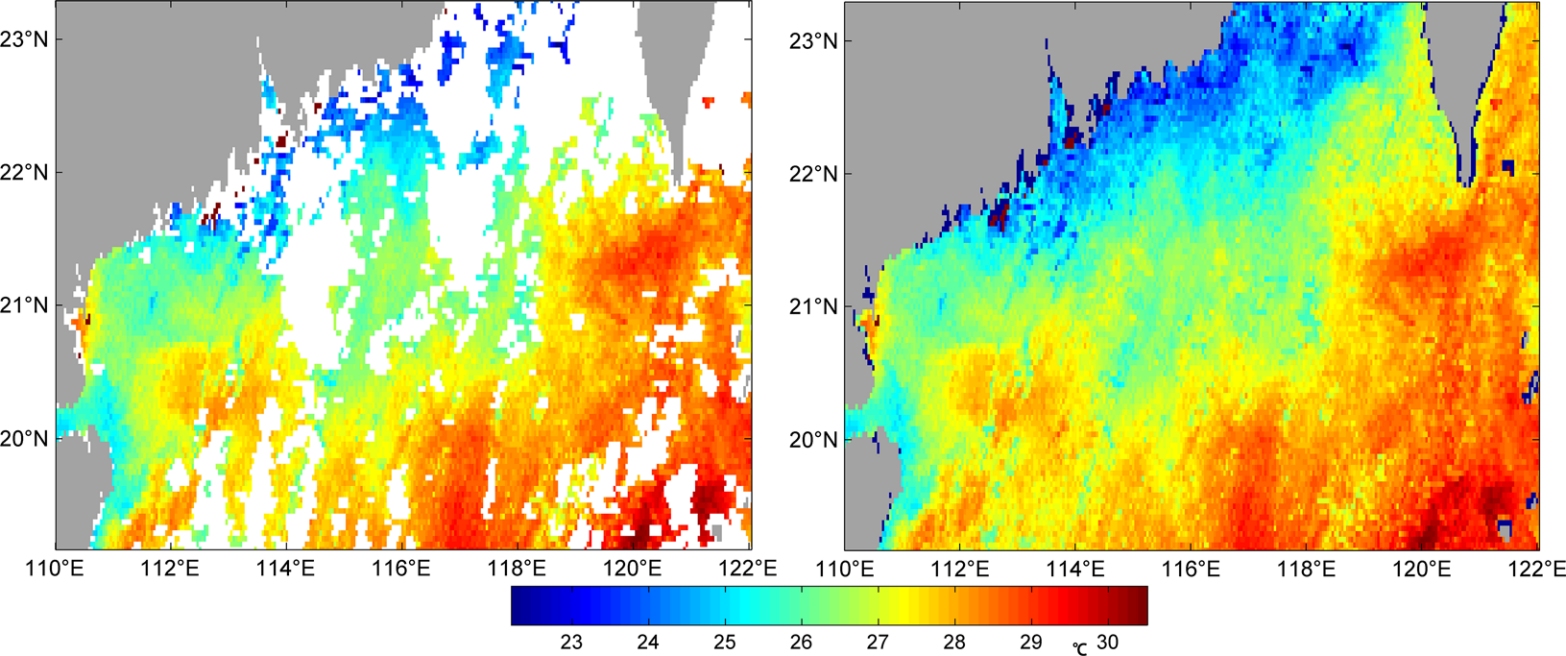
Left panel: original gappy image for 30^th^ April, 2012; right panel: its reconstruction image.

**Fig 6:**
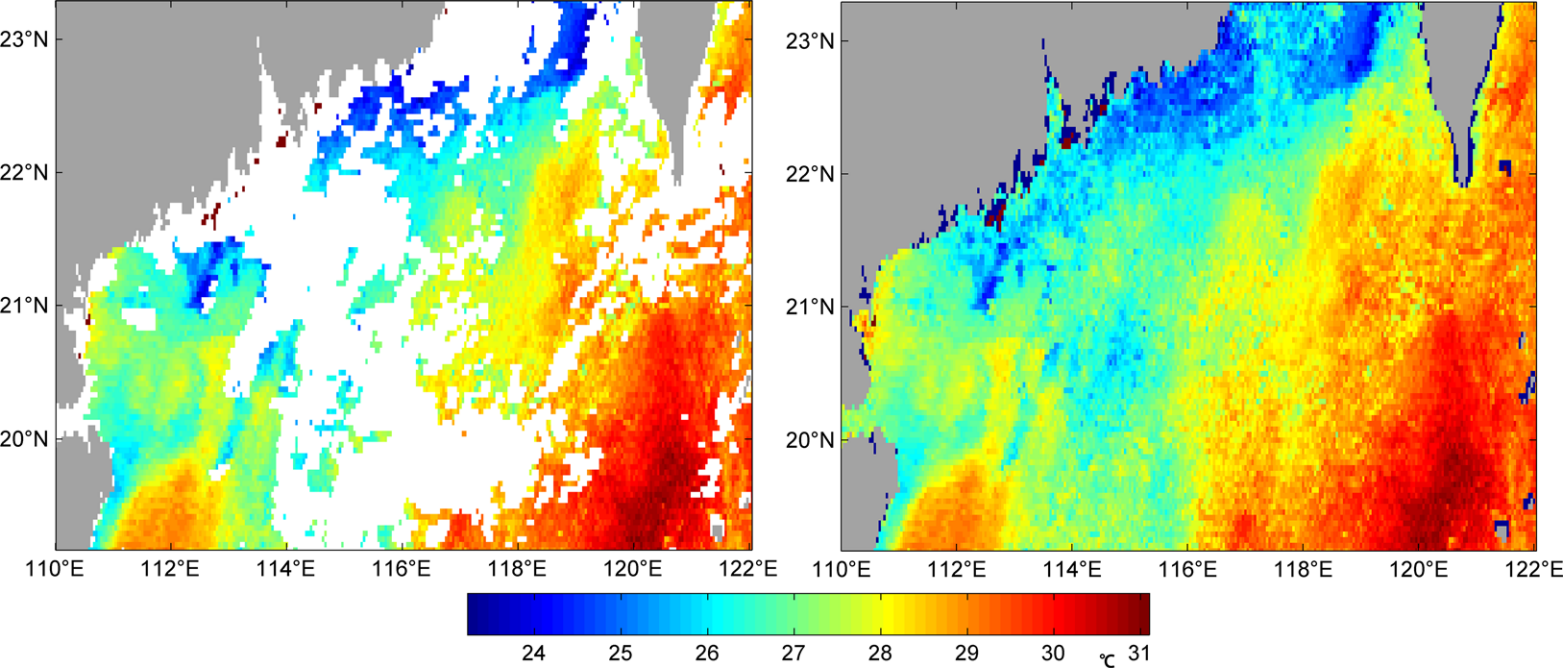
Left panel: original gappy image for 2^nd^ May, 2012; right panel: its reconstruction image.

In Figs 3–6 we can see four examples of the quality of VE-DINEOF results on the PFV5.2 SST data. They show four original images, which blanks where there are no data, and their reconstruction. Fig 3 is of 25^th^ October, 2011 which the percentage of missing data is 62.49%; Fig 4 is of 12^th^ April, 2012 which the percentage is 53.46%; Fig 5 is of 30^th^ April, 2012 which the percentage is 35.2%; Fig 6 is of 2^nd^ May, 2012 which the percentage is 42.95%. As shown in Figs 3–6, no matter what percentage of the missing data is, the VE-DINEOF algorithm can reconstruct the gappy data and fill in all missing data with reasonable values. Every reconstruction image presents a complete structure of SST and meanwhile the coherence of SST in local region is acceptable.

## Discussion

### 5.1 Optimal EOFs Determination

**Fig 7:**
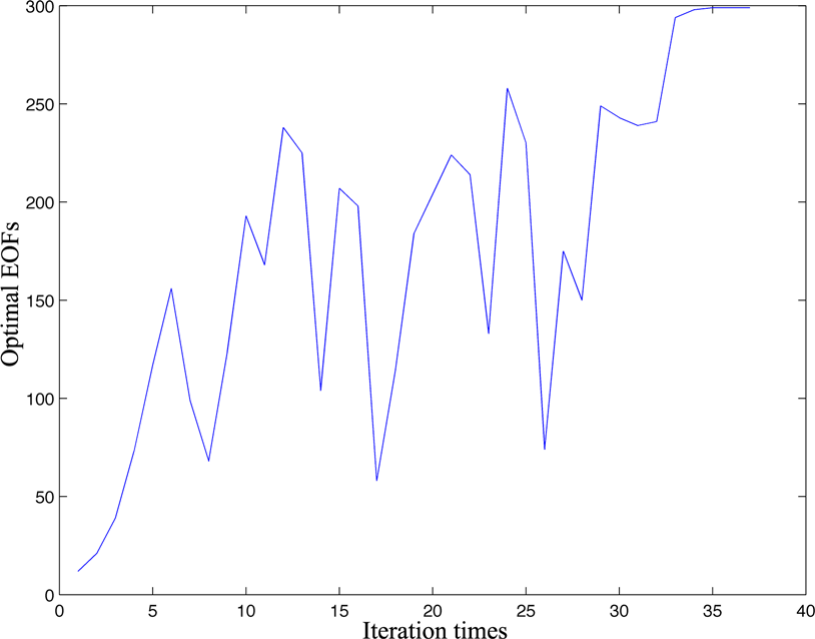
Optimal EOFs distribution in VE-DINEOF algorithm.

For the VE-DINEOF algorithm, the optimal EOFs are changeable and need to be determined based on the reconstructed matrices generated in the intermediate steps. As shown in Fig 7, the optimal EOFs distribution is irregular and the mean, maximum and minimum optimal EOFs are about 176, 299 and 12, respectively. The trend of distribution slightly increases with the increasing iteration times. On the other hand, the iteration times in total is 37 in this study, which means the convergence can be quickly reached so that the computational time is shortened.

**Fig 8:**
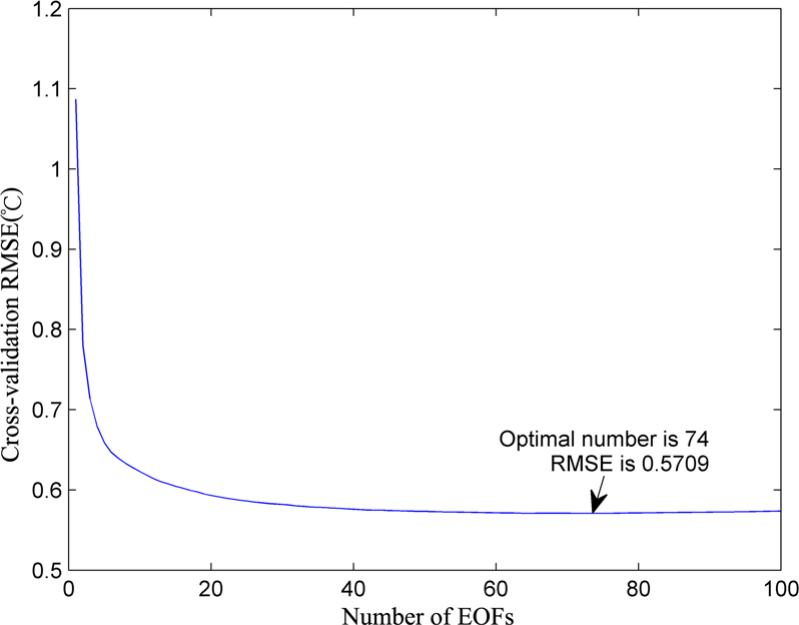
RMSEs obtained with cross-validation for reconstruction by using DINEOF algorithm.

For the same spatio-temporal matrix, the optimal EOF number in the ordinary DINEOF is 74 and the corresponding RMSE at the cross-validation points is 0.5709°C. The RMSE distribution at the cross-validation points is shown in Fig 8. We can find that the optimal EOFs number is obviously lower than the average EOFs numbers in the VE-DINEOF algorithm, which means this optimal EOF may be not the best selection for some reconstructed matrices in the intermediate steps. On the other hand, because the optimal EOF mode determination in the DINEOF algorithm is not necessary, the VE-DINEOF can significantly enhance the reconstructed accuracy.

## 5.2 Performance on Different Percentage of Missing Data

The VE-DINEOF algorithm can perform better on reconstruction than I-DINEOF and DINEOF algorithms as a whole, but we still want to know whether the VE-DINEOF algorithm can get higher accuracy than those two DINEOF algorithms for different percentages of missing data. Hence, we calculated the percentage of missing data of every image and the corresponding RMSE between original image and reconstructed image at the valid points.

**Fig 9:**
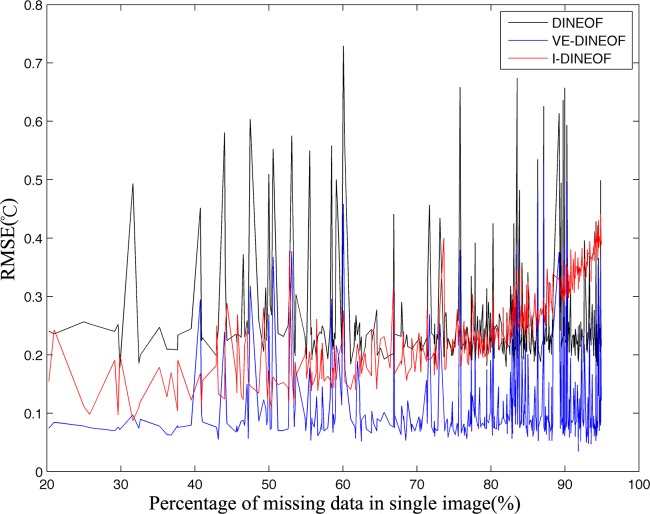
RMSEs obtained from VE-DINEOF, I-DINEOF and DINEOF algorithms for different percentages of missing data.

As shown in Fig 9, the trends of RMSEs of VE-DINEOF and DINEOF algorithms are similar, but no matter what the percentages of missing data are, the RMSEs of VE-DINEOF algorithm are much smaller than those of DINEOF algorithm. Compared with the DINEOF algorithm, the average reduction of RMSE is 0.1438°C for the VE-DINEOF algorithm. So we can confirm that the VE-DINEOF can get better results than DINEOF algorithm for different percentages of missing data.

Compared with I-DINEOF algorithm, the RMSEs obtained from VE-DINEOF algorithm are relatively small for different percentage of missing data. The trend of RMSEs of I-DINEOF algorithm seems to be smooth, which can be probably attributed to the local processing. On the other hand, the reconstructed accuracy of I-DINEOF will become worse than the other two algorithms when the percentage of missing data surpasses 90%. When the percentage of missing data is large, the existing points in some subareas may be not sufficient for optimal EOF mode determination, so the local EOF modes for reconstruction are not suitable causing the reconstructed accuracy to decrease. In some cases, we also noticed that the I-DINEOF algorithm could acquire higher RMSEs than VE-DINEOF algorithm. The size of subarea is a significant parameter in I-DINEOF algorithm and we believe if this parameter can be better determined, the performance of I-DINEOF algorithm can be enhanced.

### 5.3 RMSEs at Cross-Validation Points

**Fig 10:**
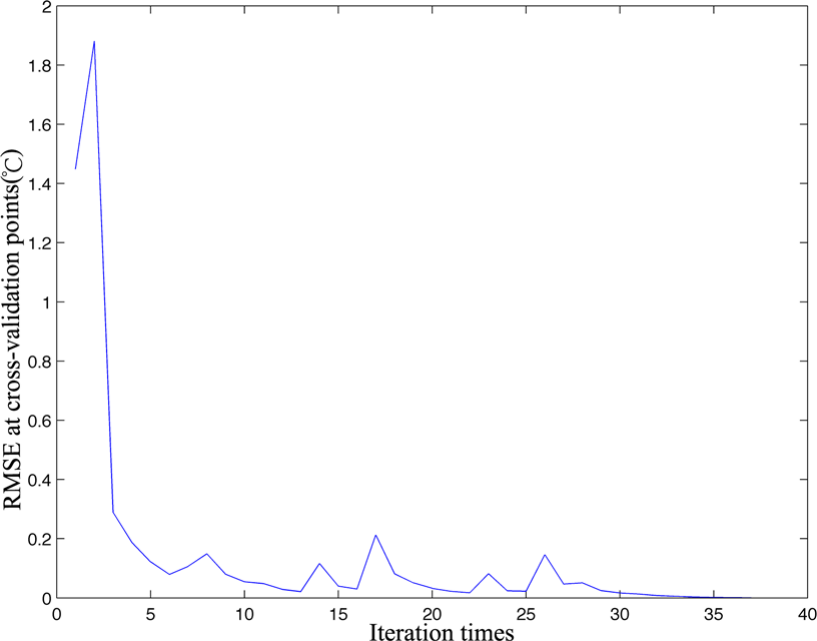
RMSEs at cross-validation points in the intermediate steps.

The convergence was defined as the RMSE between the two iterations at the cross-validation points lower than a predefined threshold in this study. The distribution of RMSEs at the cross-validation points with the increasing iteration times is shown in Fig 10. At the beginning of the iterations, the RMSE increases slightly and then steeply declines. After 5 iterations, the trend gradually becomes stable until the convergence is reached. Actually, as shown in Fig 7, at the beginning of iterations, the optimal EOF modes are comparative low, which means only a small number of EOFs are used to fill in the missing data, so, the error may become large.

### 5.4 Computational Time

The computational time depends on the specific runtime environments (CPU speed, memory capacity, program code et al.), so we did not list the specific computational time of VE-DINEOF and DINEOF algorithms. We can confirm that the VE-DINEOF algorithm can run faster about 6–7 times than DINEOF algorithm under the same circumstances. In the DINEOF algorithm, for the first EOF mode, the decomposition and reconstruction processes will be first repeated until the convergence criterion is reached. Then the EOF mode increases one by one and the whole iterations will be operated again, which means in this study, the whole iterations will be operated 100 times (the greatest number of EOFs modes in DINEOF algorithm was predefined as 100 in this study. Because the optimal number of EOFs is 74, we believe the predefinition is reliable.). However, in the VE-DINEOF algorithm, the convergence is only supposed to be reached once. This modification can apparently enhance the computational time even though the greatest number of EOF modes in the VE-DINEOF algorithm (predefined as 300) is much larger than that in the DINEOF algorithm.

On the other hand, after obtaining the optimal EOF mode in the DINEOF algorithm, the whole reconstruction process will be operated again based on the optimal EOF. But in the VE-DINEOF algorithm, this process is not necessary. Hence, the VE-DINEOF algorithm can reconstruct the initial matrix more efficiently.

## Conclusion

An improved DINEOF algorithm was presented in this study. In the VE-DINEOF algorithm, the missing data are reconstructed based on changeable optimal EOF modes instead of one fixed EOF mode and the optimal EOF modes need to be determined by using cross-validation technique for reconstructed matrices in the intermediate steps. After reconstructing by one optimal EOF mode, the missing data in the spatio-termporal matrix should be replaced by the updated values, so the spatio-temporal matrix varies after each reconstruction. Theoretically, different matrices can generate different optimal EOFs, hence, compared with reconstructing the matrix with one fixed EOF mode, the VE-DINEOF can get a better reconstructed accuracy.

On the other hand, the computational time of VE-DINEOF algorithm in total is much shorter than that of the ordinary DINEOF algorithm under the same circumstances. The reason is mainly that in the VE-DINEOF algorithm, the reconstructed repetition by using one fixed EOF mode until convergence is not necessary and the convergence only needs to be reached once. Of course, if the maximum number of EOFs can be automatically determined, we believe the efficiency of VE-DINEOF algorithm will be exponentially enhanced.

Through the experiments on AVHRR PFV5.2 SST data, the four validation parameters (r, SNR, RMSE and MAD) of the VE-DINEOF algorithm are all better than those of the I-DINEOF and DINEOF algorithms. In addition, the computational time is about 6–7 times shorter than the DINEOF algorithm.
